# ECOLOGICAL RISK ASSESSMENT IN THE CONTEXT OF GLOBAL CLIMATE CHANGE

**DOI:** 10.1002/etc.2047

**Published:** 2012-12-18

**Authors:** Wayne G Landis, Judi L Durda, Marjorie L Brooks, Peter M Chapman, Charles A Menzie, Ralph G Stahl, Jennifer L Stauber

**Affiliations:** † Western Washington University, Institute of Environmental ToxicologyBellingham, Washington, USA; ‡ Integral ConsultingAnnapolis, Maryland, USA; § Zoology, Southern Illinois UniversityCarbondale, Illinois, USA; ‖ Golder AssociatesBurnaby, British Columbia, Canada; # ExponentAlexandria, Virginia, USA; †† DuPont Company, DuPont Corporate Remediation GroupWilmington, Delaware, USA; ‡‡ CSIRO Land and WaterKirrawee, Sydney, Australia

**Keywords:** Ecosystem service, Multiple stressors, Regional risk assessment, Uncertainty

## Abstract

Changes to sources, stressors, habitats, and geographic ranges; toxicological effects; end points; and uncertainty estimation require significant changes in the implementation of ecological risk assessment (ERA). Because of the lack of analog systems and circumstances in historically studied sites, there is a likelihood of type III error. As a first step, the authors propose a decision key to aid managers and risk assessors in determining when and to what extent climate change should be incorporated. Next, when global climate change is an important factor, the authors recommend seven critical changes to ERA. First, develop conceptual cause–effect diagrams that consider relevant management decisions as well as appropriate spatial and temporal scales to include both direct and indirect effects of climate change and the stressor of management interest. Second, develop assessment end points that are expressed as ecosystem services. Third, evaluate multiple stressors and nonlinear responses—include the chemicals and the stressors related to climate change. Fourth, estimate how climate change will affect or modify management options as the impacts become manifest. Fifth, consider the direction and rate of change relative to management objectives, recognizing that both positive and negative outcomes can occur. Sixth, determine the major drivers of uncertainty, estimating and bounding stochastic uncertainty spatially, temporally, and progressively. Seventh, plan for adaptive management to account for changing environmental conditions and consequent changes to ecosystem services. Good communication is essential for making risk-related information understandable and useful for managers and stakeholders to implement a successful risk-assessment and decision-making process. Environ. Toxicol. Chem. 2013;32:79–92. © 2012 SETAC

## INTRODUCTION

Global climate change (GCC) is accepted increasingly within the scientific and regulatory communities and the informed public as capable of impacting human and ecological systems for centuries 1, 2. This report stems from a SETAC Pellston Workshop convened to assess the influence of GCC on the scientific foundations and applications of environmental toxicology and chemistry, specifically from the work group charged with determining how ecological risk assessment (ERA) needs to change to take into account GCC. Stahl et al. 3 provide a detailed account of the origins of the workshop and summarize the findings of other working groups.

At its core, ERA is a tool that is used to inform management decisions. Although the ERA process has evolved since its formal inception in the early 1980s 4, many limitations and challenges remain 5 that can decrease its effectiveness in addressing management decisions. Current ERA frameworks, such as those under the U.S. Environmental Protection Agency Superfund Program 6, were developed to examine risks from particular stressors (primarily chemical) acting on particular receptors bounded within relatively small geographic areas and to largely ignore other noncontaminant stressors (physical or biological). Consequently, with the exception of a limited number of watershed and regional assessments 7, 8, there is little experience with applying the framework to changing landscapes and multiple drivers.

## FUTURE PREDICTIONS OF CLIMATE AND THE TYPE OF ECOLOGICAL SYSTEMS UNDER CLIMATE CHANGE

A first question in applying risk assessment in a climate change scenario is, To what extent will current conditions be altered? Assumptions regarding the fate and transport of chemicals, the toxicological effects, and the resultant changes in ecological dynamics are based on current climates and ecological systems. Because data are available only for current conditions and for those sampled in the recent past, extrapolation to conditions under climate change may add to the uncertainty.

The historic range of variability of systems does provide a measure of past environmental variability in systems with adequate sampling and analysis 9. In many circumstances, however, the data required to estimate historic range of variability may not be available for specific end points. Anderson and Landis 10 found that historic range of variability data as applied to a risk assessment were not always available, even for forestlands that have been extensively studied over decades. Thus, uncertainty exists over what, exactly, those past conditions were.

A number of recent articles predict with low uncertainty that conditions under climate change will produce novel climate and ecological systems. Williams and Jackson 11 demonstrate that numbers of ecosystems will exist in the future that have no current analog in terms of their temperature and precipitation regimes. Given that climate change is likely to produce a number of no-analog climates 12, it is also likely that a number of no-analog ecological communities will result.

Milly et al. 13 point out that in the field of water management, assumptions of unchanging conditions have been overturned by a paradigm of continued change. Anthropogenic impacts on climate have already been demonstrated to change the availability of water over the world. Clearly, the past is now a poor model of the future of water resources.

Walther 14 provides additional information on the change in ecosystems due to recent climate change. One of the highlights of this review is that linear models are poor predictors of impacts. The author suggests that the linkages among the components of ecological systems are critical and that interactions and feedback mechanisms can lead to nonlinear and abrupt changes.

Recent studies have revealed issues with uncertainty in the management of valued species and with small-scale climate predictions. Predicting the management of ecological resources needs to be recognized as an evolutionary process, and the conservation of valued species needs to recognize this component 15. Issues exist with climate projections being downscaled to match those for conservation management. The uncertainty of the downscale is because of the lack of sampling points at appropriate scales 16. In the context of risk assessment, this means that considerable uncertainty in climate projections will exist at small-scale sites, which are typical with small cleanups or spills. Even risk assessments at the scale of small watersheds may have the additional uncertainty of climate predictions added to the process.

The uncertainties in the predictions of future climates and the effects on climate change can lead to a different type of error issue—type III error. A type III error is when a correct analysis is conducted but to the wrong question for establishing the cause 17. A type III error can occur when conditions are so novel that previous experience is not informative as to what question should be asked or what model constructed. As risk assessment is asked to calculate conditions further into the future where no-analog communities are more likely to exist, the likelihood of a type III error will also increase.

Given the above, we know that changes to climate exist and that these can result in no-analog ecological communities. Further prediction at a variety of scales can be problematic because of the limitations of the existing data sets and the resulting predictive models. In this context of climate change, we made our recommendations for conducting risk assessment. With GCC comes the need to develop ERAs that are broader in scope than traditional chemical risk assessments. Following are four fundamental considerations.

First, ERAs must consider interactions among contaminant and noncontaminant stressors. With new regimes of temperature and precipitation at specific geographic sites, novel ecosystems 11 with novel hydrologic processes 13 will be created that will trigger novel responses to lethal and sublethal doses of chemical stressors. Historically, environmental management decisions often relied on past information to judge what can be expected in the future. Familiar examples include using specified river-flow statistics for regulating discharges of effluents or using definitions of flood zones for guiding building codes and the design of waste-disposal systems. However, directional changes in climate undermine the reliability of historical statistics and introduce uncertainty into the information used for making environmental management decisions.

Second, changing climate requires a shift not only in the science but also in regulatory programs. For example, traditional ERAs, especially those focused only on chemicals, often rely on simple tools such as hazard quotients, which are not adequate for evaluating multiple stressors and associated interactions. These hazard quotients (defined as the ratio between observed contaminant levels and a regulatory guideline) are often used to rank risks from chemicals in natural systems. However, this regulatory criterion derives from a series of exposure–response curves that are generated with a few model species exposed in artificial waters and have minimal resemblance to natural waters. Although useful in some screening ERAs, such simplistic representations cannot fully characterize risk, nor can they characterize the full range of possible exposure responses and their associated uncertainty 18.

Third, greater emphasis on and understanding of stochasticity, tipping points, and multistressor interactions is needed. These interactions include the interactive effects of physical and biological stressors such as more frequent spikes in contaminant input because of greater storm frequency and intensity 19, 20 or greater competition for resources from invasive species 21, 22. Resource managers are well acquainted with the dynamic nature of ecosystems, recognizing that baselines are irrevocably changed with compounded perturbations (i.e., the shifting baselines concept) 23, 24. However, that construct infers that all change will be negative, when impacts from climate change will actually be both negative and positive. Therefore, managers must focus on adaptation strategies that take into account current and changing resources as affected by contaminant and noncontaminant stressors. This process needs to be embraced and expanded and can be accomplished by implementing a regional risk-assessment approach 25, 26. Regional risk assessment is structured to address multiple stressors and their impacts on multiple ecological services.

Fourth, realizing that biological responses to environmental stressors likely will be nonlinear, especially under GCC, the previous reliance on null hypothesis models needs to be discarded. Traditionally, ERAs evaluated whether there is a change in risk relative to a reference site or condition where only one variable is being considered. Because ecological conditions will change unpredictably with GCC, the interactions among variables are dynamic and response to GCC will evolve; therefore, simplistic assumptions of static conditions and unidirectional change are no longer appropriate (e.g., Rohr et al. 27).

To formalize these realizations, we propose seven principles for guiding future ERAs that will assist in providing better information for better management decisions in a GCC-influenced world. These principles and their application are illustrated by two case studies. The seven principles for conducting ERAs within the context of GCC (Table 1) were developed after considering the limitations of the current ERA frameworks. Using case studies, we demonstrate that continuing to ignore GCC and the interactions with the impacts of contaminants will greatly reduce the accuracy of ERAs and their utility in supporting technically sound and effective management decisions.

**Table 1:** The seven principles for conducting ecological risk assessment (ERA) in the context of global climate change (GCC)

Principle 1	Consider the importance of GCC-related factors in the ERA process and subsequent management decisions.
Principle 2	Assessment end points should be expressed as ecosystem services.
Principle 3	Responses of ecosystem services (end points) can be positive or negative.
Principle 4	The ERA process requires a multiple stressor approach, and responses may be nonlinear.
Principle 5	Develop conceptual cause–effect diagrams that consider relevant management decisions as well as appropriate spatial and temporal scales to allow consideration of both direct and indirect effects of climate change.
Principle 6	Determine the major drivers of uncertainty, estimating and bounding stochastic uncertainty spatially and temporally, and continue the process as management activities are implemented.
Principle 7	Plan for adaptive management to account for changing environmental conditions and consequent changes to ecosystem services.

## SEVEN PRINCIPLES FOR IMPROVED ERA AND MANAGEMENT DECISIONS UNDER GCC

### Principle 1: Consider the importance of GCC-related factors in the ERA process and subsequent management decisions

**Fig. 1:**
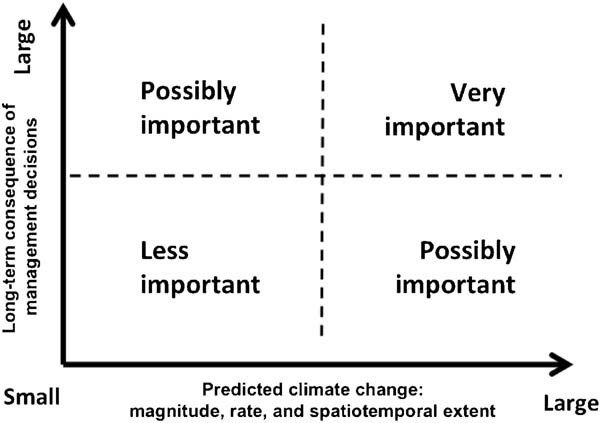
Judging the importance of considering climate change drivers for environmental risk-management decisions.

Climate change will not always be an important factor in future ERAs. An initial triage is necessary. Figure 1 displays a suggested strategy for this process starting with two attributes—degree of climate change (magnitude, rate of change, and spatiotemporal scale) and long-term consequences of the management decision—that can be used to rank the relative importance of climate when developing ERAs. Explicit consideration of climate change is most important for management decisions for geographic areas where the impact of climate change is large and the consequences of the management decisions are long-term.

To demonstrate this approach, we considered a range of management scenarios and ranked them by the degree to which climate change should be factored into the ERA process (Table 2). Stressors occurring over a short time period and across a limited space received the lowest rank; those occurring over long time periods and large areas received the highest rank. We also considered the temporal and spatial scales of management actions and their likely reversibility. For the temporal criteria, we considered <1 to 10 years as short-term, >10 to 50 years as intermediate-term, and >50 years as long-term. For spatial criteria, we considered landscapes less than a watershed as small, watersheds as intermediate, and scales above watersheds as large. For demonstration purposes, we assigned a score of 2, 4, or 6 to each criterion to rank it with respect to the likely importance of climate change in the evaluation, with a score of 2 representing low importance. In order of low to high, Table 2 lists the influence of GCC on a number of management decisions. For example, climate is unlikely to influence the cleanup of a localized fuel spill on an impermeable surface, hence the relatively low score. In contrast, many of the currently most controversial management decisions received high scores. Examples include the potential consequences of hard-rock mining, hydrofracking, and the construction of dams, all of which have great potential to be influenced by greater storm frequency and runoff of acids leached from exposed mine tailings, greater evaporation of potable surface water, and higher demands for hydroelectric power.

**Table 2:** Climate change considerations and ranking for different management scenarios

Management scenario	Stressor			Likelihood and magnitude of climate change influence on stressor		Scale and permanence of management decision	Sum/score
	
	Physical	Chemical	Biological	Temporal	Spatial		
Cleanup of localized fuel spill		x		2	2	2	6
Point source effluent discharge		x		2	2	2	6
Annual application of herbicides along right of way		x		2	4	2	8
New consumer chemical (low persistence)		x		2	6	2	10
Contaminated sediments		x		6	2	4	12
Biosolids application		x		6	2	4	12
Fishery management (harvest)			x	4	4	4	12
Chemical total maximum daily loads (high persistence)		x		4	4	4	12
Nutrient enrichment surface water		x		4	4	6	14
Water use (drinking water vs ecological flows)	x			4	4	6	14
Agricultural irrigation and salinization	x	x		4	4	6	14
Water use for oil sands	x			6	4	6	16
Hard-rock mining	x	x		6	6	6	18
Coal seam gas drilling/hydrofracking	x	x		6	6	6	18
Invasive species			x	6	6	6	18
Dam construction	x			6	6	6	18
Forest management			x	6	6	6	18

### Principle 2: Assessment end points should be expressed as ecosystem services

The relative importance of climate change on human dimensions depends on a variety of regulatory, technical, and political drivers that are subject to socioeconomic dynamics. Although the full range of the consequences of GCC cannot be predicted, it is reasonable to assume that GCC may affect human health and welfare in ways that prompt management decisions that are seemingly at odds with ensuring ecosystem structure and function. Routinely, managers will need to make decisions that balance each of these end points. As a consequence, analyses that provide outcomes for all end points in a currency, which is transparent and interpretable by all stakeholders, will help to support management decisions. Articulating all aspects of a risk assessment in terms of ecosystem services provides a useful common currency for deciding what to protect across the broad spectrum of ecosystem components and management end points.

*Ecosystem services* are defined as the products of ecological functions or processes that directly or indirectly contribute to human well-being or have the potential to do so in the future. Effectively, they embody the benefits of ecosystems to households, communities, and economies 28–31. Using ecosystem services in an ERA for estimating potential impacts on humans from management actions is becoming increasingly important 32–34.

Framing ERA and subsequent management decisions in terms of ecosystem services allows for the consideration of trade-offs (i.e., risk–risk comparisons). For instance, rising sea levels, which increase salinity in near-shore freshwater environments, may ameliorate the toxicity of some metals and thereby reduce risks from contaminated sediment to the natural system. Making management decisions under this situation would be difficult given that both scenarios (increased salinity and contaminated sediments) have outcomes that would commonly be considered adverse. The use of ecosystem services as an analysis metric could place the outcomes on a common scale and facilitate decision making 35.

Industries and governments are investing in approaches that rely on the characterization of ecosystem services, and these initiatives can be further developed to address the types of ecological risk questions being raised in the context of climate change. Businesses for Social Responsibility (BSR) provides a forum for exploring how to apply ecosystem service concepts in the business world. In 2007, BSR launched a working group to examine this issue. In its first year, the working group developed a guide for corporate managers to the emerging risks and opportunities associated with corporate reliance on, impact to, or revenue opportunities from ecosystem services and environmental markets. Businesses for Social Responsibility continues to advance these concepts (http://www.bsr.org/files/BSR_ESTM_WG_2011_Workplan.pdf).

Among government institutions, research and development initiatives within the U. S. Department of Defense reflect a combined recognition of the necessity of addressing risks associated with climate change and the use of ecosystem services as a potential approach for informing decision making at the U.S. Department of Defense facilities. The U.S. Department of Defense manages large tracts of land and water and has multiple missions, including those involving environmental stewardship. Many of these missions can be impacted by GCC. Because such changes are regional in nature, they, as well as national and global approaches, are being considered by various entities within the U.S. Department of Defense.

Many of the U.S. Department of Defense's parallel initiatives on climate-related research as well as on the application of ecosystem services approaches have been closely aligned with management responsibilities related to ecological and environmental considerations. These initiatives are reflected in the array of projects under active investigation within the Strategic Environmental Research and Development Program/Environmental Security Technology Certification Program (SERDP/ESTCP, http://www.serdp.org/Program-Areas/Resource-Conservation-and-Climate-Change).

Military installations are safe havens for hundreds of listed and at-risk species. They encompass a wide variety of ecological systems that contribute to biological diversity. The U.S. Department of Defense recognizes that climate-related effects are already being observed at military installations in every region of the United States and its coastal waters. Current areas of emphasis for climate-related ecological and environmental impacts include understanding U.S. Department of Defense–relevant ecological systems that are considered to be at particular risk (coastal areas, arctic, and arid areas in the southwest), managing threatened, endangered, at-risk, and invasive species, and developing the models and tools necessary to understand infrastructural vulnerabilities to climate change and the resultant impacts.

### Principle 3: Responses of ecosystem services (end points) can be positive or negative

The term *risk*, defined only as the probability of an adverse outcome, is not adequate to capture the range of ecological outcomes that may occur with ongoing climate change. Climate changes can result in increases as well as decreases in the amount and quality of habitats, greater viability of populations of species, and enhancement of various ecological functions 36. For example, in some cases, climate change will extend the range of land suitable for agriculture; it will also facilitate invasion by nonindigenous species, which can have both positive and negative impacts when evaluated from a number of perspectives. Therefore, analyses that are rooted in the assumption of unidirectional and adverse change as a result of GCC will be erroneous.

### Principle 4: The ERA process requires a multiple-stressor approach, and responses may be nonlinear

**Fig. 2:**
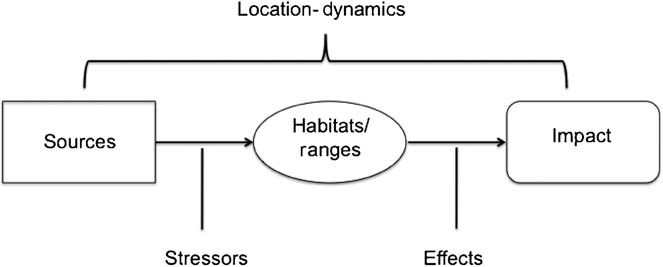
Cause–effect diagram for risk assessment under climate change. As climate change becomes manifest, the spatial relationships of the various factors can change along with the temporal dynamics. The causal pathways may also change depending upon changes in physical habitat and the species composition of the system.

The very nature of climate change is that it is a multistressor process. Therefore, given changing climate, ERAs must consider multiple stressors and the assessment methods must be appropriate for that purpose. Methods for assessing multiple stressors include the driver-pressure-state-impact-response approach 37, which has been applied to watersheds 38, as well as watershed-based approaches in, for instance, the United States 39. A regional and multiple-stressor approach to risk assessment is the relative risk model 25, 26, 40. Figure 2 illustrates the basic formulation of the cause–effect pathway used as the foundation of the relative risk model. Note that there are a variety of sources of multiple stressors interacting at a number of locations with various ecological components. Global climate change acts as another source with another suite of stressors operating on the landscape.

Responses to stressors can be nonlinear, with tipping points and nonlinear dynamics rather than dynamically simple, linear changes. Tolerance (both physiological acclimation and evolution) needs to be considered. Finally, a much greater level of stochasticity must be recognized as a reality; with changing climate, the environment will be changing globally and background or baseline data will be useful only for tracking the trajectory of change relative to ecosystem services 41.

Timescales are of great importance, particularly integrating information on rates and magnitudes of climate-related changes with the various timescales of ecological processes and the temporal characteristics of management decisions (Fig. 1). Global climate change will alter habitats and shift to nonanalog systems that do not have the previously optimal temperature and moisture ranges for local populations. This shift will trigger tolerance and/or prompt movement of the species to more favorable geographic areas when possible. Although presently there is limited understanding of rates and magnitudes of change in response to changes in climate, explicit consideration and integration of temporal scales is likely the most important aspect of formulating an approach for ERA that takes climate-related changes into account.

The nature of the interaction of climate change with chemicals and other stressors requires a variety of approaches that depict the probabilistic nature of the system and articulate causes and effects as probabilities. This will be a departure from more traditional approaches that rely on simpler deterministic metrics. Such an approach is Bayesian networks.

Analyses dealing with spatial and temporal scales and nonlinear dynamics may benefit from the use of Bayesian networks, which incorporate probabilistic relationships and explicitly deal with uncertainty. Bayesian networks are graphical models that use conditional probability distributions to describe relationships between model variables. This approach has been applied to ecological systems 42–46. Using Bayesian networks can reduce uncertainty in a model due to a lack of knowledge because different types of information, including model predictions and expert judgment, can be used as data input. Bayesian networks are inherently hierarchical and causal, making them a valuable tool for evaluating alternative management strategies and assessing the synergistic effects of various disturbances.

### Principle 5: Develop conceptual cause–effect diagrams that consider relevant management decisions as well as appropriate spatial and temporal scales to allow consideration of both direct and indirect effects of climate change

Conceptual diagrams need to encompass sources, stressors, pathways, and receptors in the context of relevant management options, as well as appropriate spatial and temporal scales. In particular, they cannot be simply snapshots-in-time, rather, they need to consider time-series changes in exposure and effects as well as cover broad enough spatial scales to consider climate-related direct and indirect effects. Key questions that need to be addressed include the following: What are the ecosystem services, and are they supported by sufficient quantitative data? Where are they located, and will their value change with GCC? What are the management options? How long and to what extent can the system be managed? How can the system best be managed given the available tools and scenarios?

**Fig. 3:**
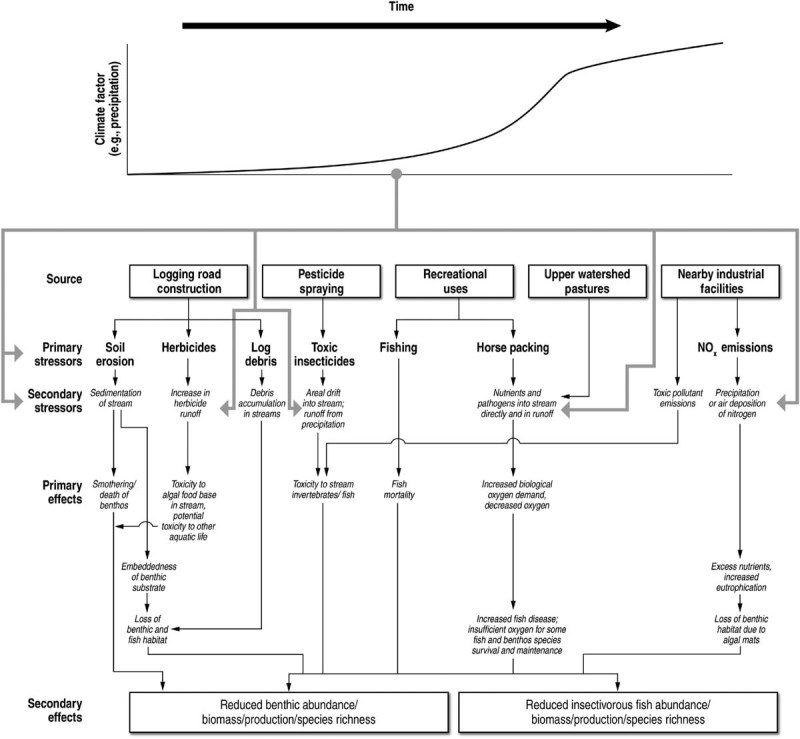
Climate change and conceptual model construction. This diagram provides an example in which time and an associated climate change factor are included in a multistressor conceptual model. Gray arrows point to those parts of the conceptual model directly affected by the changing climate variable.

Most ERAs begin with a conceptual model as a critical part of problem formulation. These have evolved from simple models for single stressors to more complex multistressor models. The explicit consideration of climate change brings with it the challenge of representing temporal and spatial changes in key drivers such as precipitation, temperature, hypoxia, and extreme events. There are a variety of ways to combine temporal considerations into a conceptual model, and these will need to be explored for various applications and audiences. Figure 3 provides an example in which time and an associated climate change factor (precipitation in this case) are included in a multistressor conceptual model.

**Fig. 4:**
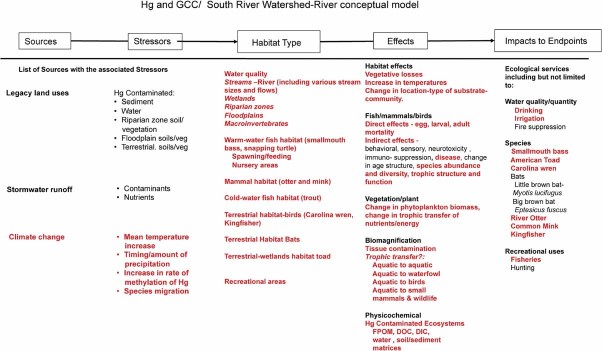
South River conceptual model example with climate change. Two sources are included, with multiple stressors from each. Locations of sources, stressors, habitats, effects, and impacts are mapped for this specific site. Words that are red and bold are those items affected by climate change.

An example of a cause–effect conceptual model is presented in Figure 4 and is based on the South River (Virginia, USA). This site is undergoing restoration because of the legacy use of mercury in the manufacture of synthetic fibers from 1928 to the early 1950s as well as other chemical inputs. This example is illustrative because of the variety of stressors, habitats, and ecological services involved and because climate change was also considered (e.g., increases in temperature, changes in precipitation patterns, and rate of the methylation of mercury). Six risk regions were defined, depending on the types of sources, land use, and in-stream sampling locations. The management scenarios include best management practices to reduce inputs of nutrients and/or Hg as well as restoration of the riparian zones. Figure 4 also incorporates the extent of the effect of climate change on ecological services and their components.

### Principle 6: Determine the major drivers of uncertainty, estimating and bounding stochastic uncertainty spatially and temporally, and continue the process as management activities are implemented

As noted in principle 5, above, uncertainty of all types will increase with GCC, and nonlinear changes, including tipping points, will occur and must be considered. Although uncertainties related to some of these events cannot be reduced beyond certain limits, they need to be estimated and bounded spatially, temporally, and as management activities occur, to allow for adaptive management (*principle 7*, below).

Uncertainty can be described by a number of methods. Monte Carlo methods have been extensively used in risk assessment, and tools such as P-bounds have also been developed. Bayesian networks easily incorporate a number of types of uncertainty into their formulation.

It is important to recognize that there is uncertainty associated with the stressors from GCC. The GCC models have a number of connected uncertainties dealing with time frame and spatial scales. It is critical to describe these uncertainties in the conceptual and cause–effect models of the risk assessment.

### Principle 7: Plan for adaptive management to account for changing environmental conditions and consequent changes to ecosystem services

Considerable uncertainty is associated with forecasting changes associated with GCC and with identifying the most appropriate management actions. These inherent uncertainties argue for an adaptive management approach. Adapting management actions and interventions to changing environmental conditions and to consequent potential changes to ecosystem services will help to address the following three potential factors that can hinder the effectiveness of assessment and subsequent management actions 47: (1) the wrong drivers (e.g., politics rather than good science), (2) a poor initial design of the process, and (3) a lack of clarity regarding protection goals and components of the ERA.

## RISK COMMUNICATION

One of the unique issues with GCC is that communication is at the forefront of public debate. Although not included in our seven principles of the risk-assessment process, it stands out as an overriding issue in connecting the risk assessment to policy making.

Reynolds et al. 48 conducted an extensive survey of what stakeholders know about GCC. His respondents were relatively well educated: 93% had graduated from high school, 43% had graduated from college, and 25% had some graduate training. Using the same survey instrument, Reynolds et al. compared their 2009 survey to results they obtained from a similar research survey conducted in 1992. Compared with the 1992 respondents, the respondents in 2009 had a greater knowledge of the specifics of GCC. However, and in spite of a great deal of coverage, it was found that many individuals did not understand that such critical relationships are because of an increase in CO_2_ in the atmosphere and that the predominant source is the burning of fossil fuel.

A survey conducted in 2010 by the Yale Project on climate change 49 demonstrated the range of beliefs regarding GCC. When respondents were asked if they thought that climate change is happening, 57% said yes, 20% said no, and 23% were not sure. The percentage of respondents who thought that climate change is happening decreased by 14 percentage points from a similar survey in 2008. (The survey had 95% confidence limits of ± 3%.)

Based on the results of the above survey, Summerville and Hassol 50 proposed that a better job be done in communicating the science of climate change to the general public. Summerville and Hassol point out that scientists often fail to make simple, clear, and accurate messages for the general public. One of the clearest issues is the specific terminology that we use as scientists and the meaning among the public. Two examples are the terms *manipulation* and *scheme*. *Manipulation* to scientists generally means that the data have been processed to search for patterns. As pointed out by the authors, *manipulation* has the general meaning of illicit tampering of information for a specific bias. To scientists, *scheme* typically means a well-thought-out and systematic plan, while *scheme* in the vernacular means devious plot—not what we as scientists intend to communicate.

Risk assessors, even in classical scenarios dealing with contaminants, invasive species, or other applications, have challenges in communicating to decision makers and stakeholders. Some of these difficulties also occur in the communication of GCC issues. It is important that toxicologists and especially risk assessors improve their ability to communicate difficult ideas to a broader constituency.

## CASE STUDIES

To illustrate the above seven principles, two case studies are described below. The first is a hypothetical case study, in which potential future climate change stressors are superimposed on an existing impacted estuary, the San Francisco estuary (California, USA). The second case study is an example of a recent water quality–screening risk assessment in the drought-impacted Murray River system in South Australia. These case studies specifically illustrate each of the seven principles.

### Case study 1: San Francisco estuary

**Fig. 5:**
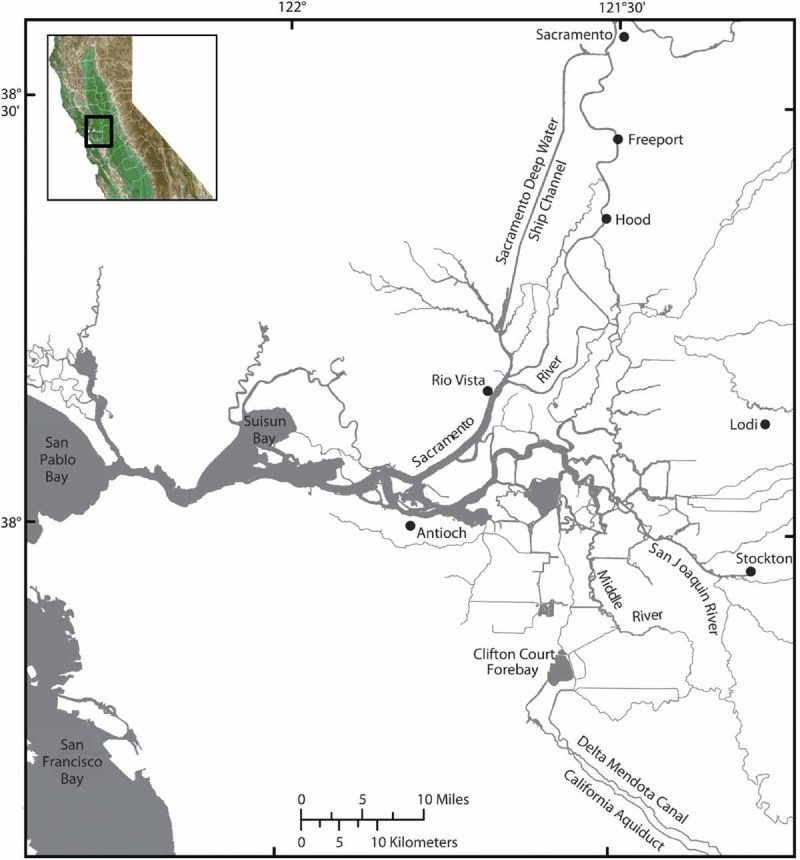
The San Francisco Bay estuary region, adapted from Brooks et al. 56. [Color figure can be seen in the online version of this article, available at http://wileyonlinelibrary.com]

The San Francisco estuary encompasses San Francisco Bay, the brackish Suisun Bay, and the freshwater Sacramento–San Joaquin River delta (Fig. 5). Water withdrawals and reduced water quality that accompany intensive urban and agricultural development around estuaries are commonly associated with major shifts in species composition 51, 52. The San Francisco estuary is no exception, having experienced rapid declines in several fish species 53.

#### Principle 1: Consider the importance of GCC-related factors in the ERA process and subsequent management decisions

Climate change is expected to greatly impact long-term management decisions for the estuary. Climate models project increased frequency of severe storms, extended drought, and 2 to 6°C increases in air temperatures in California by 2100 54. Salinity and turbidity will be altered by climate change 55, with consequent direct effects on fish populations and indirect effects on their prey. Storm patterns could increase the frequency of contaminant pulses from overland runoff by 10- to 100-fold 19, 20. Reduced summer flows (i.e., less dilution) and evaporation may increase chemical concentrations in the estuary. More importantly, less freshwater outflow will allow movement of brackish waters into historically freshwater reaches of the delta, affecting the physical size of habitat for the delta smelt (*Hypomesus transpacificus*), a freshwater obligate which prefers salinities of ≤2 psu (2,000 mg salt/L). Warmer water temperatures may increase or decrease the toxicity of some chemicals (multiple-stressor interactions, increases in the bioavailability of chemicals and metabolic rates) 56. Higher water temperatures will limit oxygen saturation and may lower feeding for the energy reserves that fish require to depurate contaminants.

#### Principle 2: Assessment end points should be expressed as ecosystem services

Approximately 25 million Californians and 12,000 km^2^ of agricultural land (annual revenues of approximately US$15 billion) rely on water exports from the delta 57. The management goal is to restore the abundance of anadromous, brackish, or freshwater fishes in the San Francisco estuary, while maintaining other ecosystem services to urban and agricultural stakeholders.

#### Principle 3: Responses of the ecosystem services (end points) can be positive or negative

Without any change in water chemistry, warming water temperatures can convert sublethal concentrations to lethal levels or vice versa. For ectotherms, warmer waters will increase respiration and feeding rates, increasing their rate of uptake of chemicals, and will increase depuration rates or lower the neural response to neurotoxins (which are greater at low temperatures). The response to higher dose varies, depending on whether enzymatic breakdown or elimination can keep pace with uptake and whether contaminants are metabolized to more toxic forms. For example, faster metabolic transformation to more toxic forms increases the toxic effect of some compounds, such as chlorpyrifos 58. In contrast, because cold slows enzymatic breakdown, some pyrethroids are exponentially less toxic at higher temperatures 59.

#### Principle 4: The ERA process requires a multiple-stressor approach, and responses may be nonlinear

Multiple stressors that affect this system include pesticides and herbicides, metals and organic contaminants from industries, agricultural runoff, and daily inputs of more than 1 billion liters of wastewater effluent, two-thirds of which do not undergo advanced secondary treatment to remove nitrogenous nutrients 60. Hydrologic changes to this system are of an adequate magnitude to reverse flow in the San Joaquin River for several months of the year. Invasive species that compete for resources, such as the bivalve *Corbula amurensis*, have greatly changed some food webs in the area 61. During every summer since 1999, portions of the estuary have experienced massive blooms of a harmful cyanobacterium, *Microcystis aeruginosa* 62, with the exception of 2010, when conditions were abnormally cool (P. Lehman, Department of Water Resources, Sacramento, CA, USA, personal communication).

**Fig. 6:**
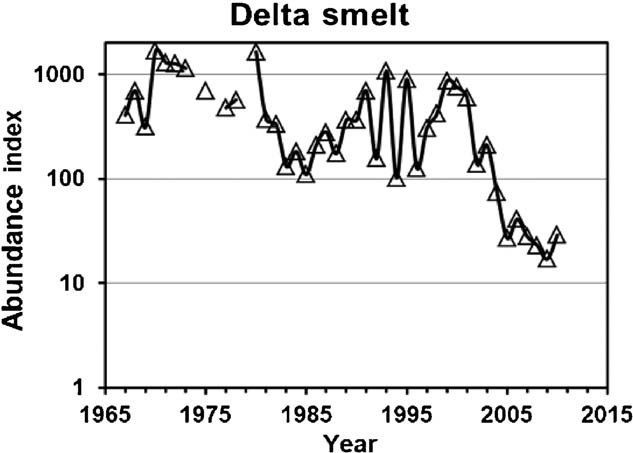
Example of a fish decline in San Francisco estuary. The numbers of delta smelt are a fraction of their historical abundance in the region. Data source: California Department of Fish and Game, http://www.dfg.ca.gov/delta/data/fmwt/indices.asp, accessed June 22, 2012.

In approximately 2002, long-term declines in the abundance of multiple fish species in the estuary accelerated abruptly, even though surrounding land use was stable and several years of above-average precipitation were expected to improve the reproductive success of many fish species 53, 56, 63. Declining species included two endemics, the delta smelt, which is listed as endangered under the United States and California endangered species acts, and longfin smelt (*Spirinchus thaleichthys*), which is listed as threatened under the California Endangered Species Act (Fig. 6). Similarly, numbers of young of the year (age 0) of striped bass (*Morone saxatilis*) and threadfin shad, which were introduced to the estuary in the late 1800s, also are declining steeply.

#### Principle 5: Develop conceptual cause–effect diagrams that consider relevant management decisions as well as appropriate spatial and temporal scales to allow consideration of both direct and indirect effects of climate change

It is hypothesized that high temperatures and nutrients that triggered cyanobacterial blooms and possibly increased use of pyrethroid pesticides could, in concert, be responsible for the decline of pelagic fishes in the San Francisco estuary 64–66. However, definitive evidence of cause and effect from contaminants remains elusive and is subject to the development of new approaches to quantify the effects of contaminants within the mosaic of multiple stressors 56. Predictive power is low and uncertainty is high because toxic effects vary within the same sampling location at different times, depending on season, urban or agricultural use of pesticides, and precipitation patterns 66, 67.

#### Principle 6: Determine the major drivers of uncertainty, estimating and bounding stochastic uncertainty spatially and temporally, and continue the process as management activities are implemented

An example of how interacting and indirect effects of climate change should be included in the analysis is demonstrated by the multiple effects of warmer waters. Some fishes require cool night temperatures to offset thermal stress 68, 69. To cope with hypoxia, fish make several physiologic adjustments, beginning with increasing their basal metabolism. Eventually, they shift to time-limited anaerobic metabolism, building high tissue concentrations of organic acids (e.g., succinate) and reactive oxygen species 69. Food limitation speeds this process because diverting blood supply to meet greater antioxidant demand burns calories 70. Greater energy is also needed to depurate contaminants 71; therefore, warmer, hypoxic, and contaminated waters will have greater detrimental effects on fishes than do cooler contaminated waters. These impacts are readily predicted using the Arrhenius equation 72 but have been tested in natural waters with endemic species only a few times 73, 74. In those studies, seasonality played an important role because warming was somewhat beneficial in winter so long as fish were not food-limited. In summer, warming coupled with high ammonia exposure was detrimental regardless of high food availability. Thus, interacting effects of temperature and contaminants introduces additional uncertainty.

#### Principle 7: Plan for adaptive management to account for changing environmental conditions and consequent changes to ecosystem services

Management options for the estuary entail mostly hydrologic, pesticide, and wastewater management, sometimes determined by court order 75. Under the dynamics of changing precipitation regimes and warming temperatures, biologic responses to seasonal patterns of water quality and quantity will vary among species depending on trade-offs between decreased toxicity of some contaminants and greater susceptibility to other contaminants, the timing and magnitude of chemical uses, storm-related spikes in exposure, and predator–prey interactions. Such uncertainty means that structured monitoring and a schedule of reevaluating management (i.e., adaptive management) are essential to the success of restoring the abundance of pelagic fishes in the San Francisco estuary.

### Case study 2: Screening ERA of the River Murray, the lower lakes and wetlands, South Australia

**Fig. 7:**
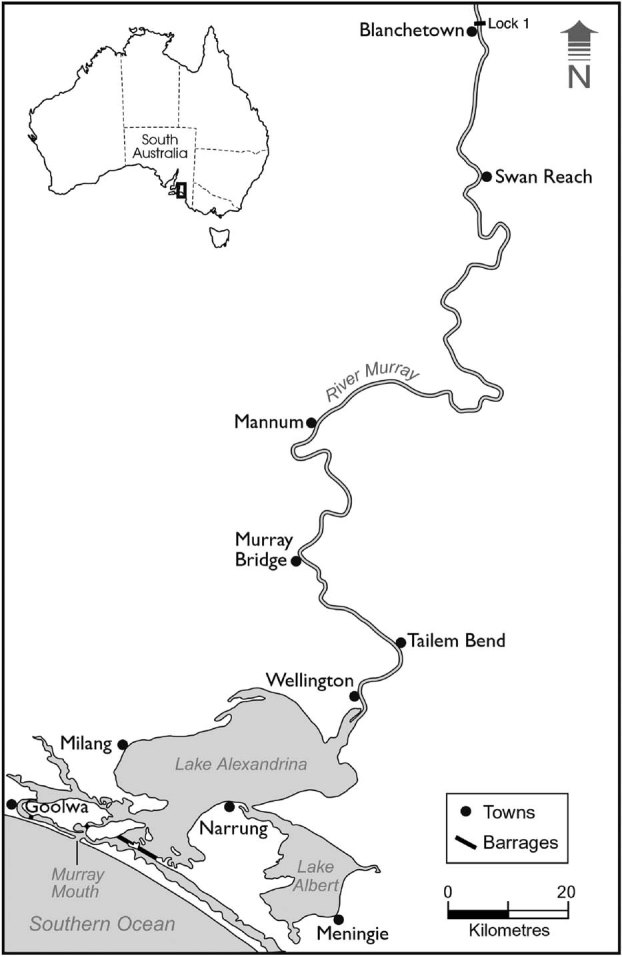
A map of the Lower Murray River System, South Australia.

The River Murray, adjacent wetlands, and the lower lakes (Alexandrina and Albert), close to the Murray mouth in South Australia (Fig. 7), have been classified as wetlands of international importance under the Ramsar Convention because of their unique ecological and hydrological significance 76.

#### Principle 1: Consider the importance of GCC-related factors in the ERA process and subsequent management decisions

Climate change is a potentially important factor in management decisions for the river. Prolonged drought compounded by over allocation of water upstream has resulted in very low water flows since 2005. These water bodies are also being impacted by both sulfidic and sulfuric materials in acid sulfate soils (ASS) 77. These ASS (pH <4) either contain sulfuric acid or have the potential to form sulfuric acid when exposed to oxygen in air through disturbance or drying. As the sulfuric acid percolates through the soils and sediments, it can leach metals into adjacent water bodies. In addition, some wetlands adjacent to the River Murray contain monosulfidic black ooze that can consume oxygen from surface waters. Rewetting of the soils and sediments from rainfall events or increased river flows (postdrought) can lead to the mobilization of acidity and of contaminants. Positive and negative effects from rainfall illustrate principle 3 (responses of ecosystem services [end points] can be positive or negative).

#### Principle 2: Assessment end points should be expressed as ecosystem services

To better understand the risks associated with rewetting of these ASS and to provide recommendations for appropriate management options, a screening ERA was undertaken 78. Three key ecosystem services were identified as follows: (1) economic—commercial fishing, tourism, drinking water, and dry land and irrigated agriculture (including dairy farming, horticulture, viticulture); (2) social and recreational—fishing, boating, swimming, and bird watching; and (3) environmental—the intrinsic value of the aquatic ecosystem including flora, fauna, and habitat.

#### Principle 4: The ERA process requires a multiple-stressor approach, and responses may be nonlinear

Materials leached into aquatic environments from ASS can produce precipitates that can alter benthic communities by smothering or altering water clarity and ultraviolet penetration 78, 79. Stress caused by oxidation products can produce longer-term effects such as decreased hatching of fish, gill disease, changes in plankton composition, and decreased biodiversity 81, 82. Occasionally, catastrophic events such as mass fish kills have been attributed to ASS 83.

#### Principle 5: Develop conceptual cause–effect diagrams that consider relevant management decisions as well as appropriate spatial and temporal scales to allow consideration of both direct and indirect effects of climate change

Conceptual models that link the major stressors and receptors were developed. Risks to aquatic biota from stressors including metals, acidity, major ions, and nutrients released from dried soils that had been rewetted via rainwater or River Murray water were determined. Predicted environmental concentrations, with correction for background water-quality data, were calculated from data on the release of metals for the soils in laboratory leachate experiments 84 for current water levels (approximately –0.5 Australian height datum) for the River Murray channel, four wetlands, and Lakes Alexandrina and Albert. Several acute pulse and chronic exposure scenarios were considered, including calculated worst-case dilutions based on the surface area of exposed sediments and river or lake volume. For the chronic scenarios, complete mixing of the acidic porewater into the lake or river following a substantial rain event was assumed, and dilutions were calculated assuming the rain event resulted in either 0.5, 1, or 2 cm water depth over the dried ASS sediment. For the wetlands adjacent to the River Murray, a simplified conceptual model for the rewetting of cracked sediments assumed that a substantial rain event would result in filling of the cracks and sediment pores with water containing contaminant concentrations similar to those found in metal mobilization elutriates (no dilution; i.e., an acute pulse exposure). Knowing the approximate surface area of each of the wetlands, the total volume of overlying water (assuming depths of 0.5, 1, and 2 cm initially) was determined.

These predicted environmental concentrations, with their associated uncertainty, were compared to predicted no-effect concentrations derived from hardness-corrected water-quality guidelines, both acute and chronic, to determine a predicted impact for each stressor. An overall risk ranking for each stressor at each location was determined, based on the likelihood and consequence of effects. Hazard quotients were also determined for two future water-level scenarios: –1.0 and –1.5 m Australian height data, assuming continuous drought conditions.

Using a range of acute and chronic exposure scenarios, the screening ERA of contaminant release from rewetted ASS to aquatic systems in the lower Murray showed that metals and acidity were ranked as high to very high risk, while nutrients, salinity, and major ions were ranked as low risk 78. The risk applied to ecosystems, public health, drinking water supplies, local infrastructure, and stock grazing 77.

Some evidence showed that the secondary stressor (low dissolved oxygen) may impact aquatic systems exposed to rewetted ASS. This may be further exacerbated by high inputs of organic carbon, which could increase the biological oxygen demand and contribute to decreased dissolved oxygen concentrations in River Murray waters and wetlands. However, it is expected that increased flow following rain events will provide some reoxygenation of these waters, and low dissolved oxygen is less likely in the lower lakes.

#### Principle 6: Determine the major drivers of uncertainty, estimating and bounding stochastic uncertainty spatially and temporally, and continue the process as management activities are implemented

The screening ERA had a number of underlying assumptions, which contributed to the uncertainties associated with both the exposure scenarios (calculated predicted environmental concentrations) and the derived hazard quotients: (1) use of worst-case ASS drying and rewetting scenarios to measure metal, nutrient, and acidity release; (2) use of upper 95% confidence limits for metal-release data and background concentrations; (3) use of worst-case dilution scenarios based on estimated rainfall and lake volumes; (4) ignoring the buffering capacity of the lake waters and river waters; (5) ignoring removal processes caused by neutralization and precipitation; (6) assuming all flow was surface flow and not considering subsurface flow; (7) not taking into account the bioavailability and interactions of metals; (8) ignoring alteration of the solubility of metals by pH; and (9) uncertain potential effects of metal precipitates on benthic biota.

#### Principle 7: Plan for adaptive management to account for changing environmental conditions and consequent changes to ecosystem services

Despite the above uncertainties, this screening ERA provided the first semiquantitative assessment of risks to aquatic biota associated with rewetting of ASS following climate change–induced drought in the iconic River Murray, adjacent wetlands, and lower lakes. Following this screening ERA, an acidification event did occur in the nearby Currency Creek following autumn rainfall in 2009. This resulted in the development of highly acidic pools with pH <3, followed by acidic flows in which approximately 700 t of acidity were mobilized 85. Several management options were considered, including liming to raise pH, revegetation, closing off selected wetlands from the river system, dredging, constructing temporary weirs, and flooding the lower river system and lakes with seawater to keep ASS submerged. The South Australian Environment Protection Authority responded by instigating a program of liming and revegetation and by constructing two temporary flow regulators to prevent the flow of acid water reaching farther downstream and to keep the ASS submerged to reduce drying and oxidation. This had some unintended consequences on the aquatic ecosystems, reducing the connectivity of the system and leading to an explosion in the population of non-native carp 86, 87, again illustrating principle 3 (responses of ecological services [end points] can be positive or negative).

Following rainfall events in mid-2010, a recommendation was made to remove the temporary flow regulators 87 and to continue a monitoring program for pH, alkalinity, and selected dissolved metal concentrations in surface waters at specific sites. It is expected that these management actions, coupled with biological monitoring and direct toxicity testing of ASS-impacted waters, should help to refine estimates of future risks associated with climate change and ASS in the lower Murray region.

## CONCLUSIONS AND RECOMMENDATIONS

We present the implications for conducting a risk assessment that calculates risk under a changing climate. As demonstrated in Table 2 and in Figure 1, many of the most controversial issues in environmental management fall into the classification where consideration of climate change is important. By our analysis, water use, hydrofracking, invasive species, oil sands, mining, and forestry all require the inclusion of climate change into the decision-making process, by the nature of their long-term potential impacts to ecological services.

We proposed seven principles that should be incorporated into risk assessments. We do not find a fundamental scientific hurdle to incorporating any of these principles into ERAs but recognize that incorporating them into existing regulatory programs in developed and developing nations will be difficult and potentially time-consuming. For each principle there is an extensive literature documenting the application. We present two case studies as illustrations, but numerous others were found to incorporate these principles or had developed appropriate methodologies for their application. Our fundamental recommendation is that the seven principles and the decision tools that we propose be adapted to decisions where climate change will play a part. Failing to include these principles will impair the efficacy of an ERA, costing managers and stakeholders time, money, and effort. In decision-making scenarios where climate change will play little or no role, the decision tools in Table 2 and in Figure 1 allow straightforward documentation of where and whether or not all seven need to be applied. In cases where climate change will be a source of stressors, all seven should be applied.

Our collective international experience also informs us that these principles and recommendations will not take place without two concurrent activities. First, decision makers responsible for optimizing their environmental management, whether in the private or the public sector, will need to include these seven principles in their ERAs whenever appropriate. Not incorporating these seven principles potentially prevents managers from informed and scientifically defensible decisions, ultimately rendering their risk assessment inadequate for practical use in decision making.

Second, risk-assessment case studies published in the peer-reviewed literature must meet the same standard. Not incorporating the seven principles in a GCC/ERA report demonstrates a fundamental misunderstanding of the scope of climate change and the potential impacts on ecological services.
